# Gaucher disease: the hematologist’s perspective of a multisystemic disorder

**DOI:** 10.1007/s00277-026-06977-3

**Published:** 2026-05-20

**Authors:** Alessandro Costa, Olga Mulas, Giovanni Caocci

**Affiliations:** 1 Department of Medical Sciences and Public Health, University of Cagliari, Cagliari, 09121 Italy; 2 Hematology Unit, Businco Hospital, ARNAS Brotzu, Cagliari, 09121 Italy

**Keywords:** Gaucher disease, Hematology, Pathogenesis, Cytopenias, Monoclonal gammopathy, Diagnosis

## Abstract

Gaucher disease (GD) exemplifies how a single genetic mutation can give rise to a complex multisystem disorder with profound hematological implications. Central to its pathophysiology is the lysosomal accumulation of glucosylceramide due to deficient glucocerebrosidase activity, together with abnormal folding and trafficking of the enzyme that induce endoplasmic reticulum stress and cellular dysfunction. These processes disrupt reticuloendothelial homeostasis and interfere with hematopoiesis. As a consequence, macrophage activation and chronic inflammation contribute to the cytopenias, splenomegaly, and hyperferritinemia that frequently lead patients to hematological evaluation. Despite significant therapeutic advances, GD remains under-recognized in routine hematology practice, often resulting in diagnostic delays and suboptimal management. The introduction of enzyme replacement therapy (ERT) and substrate reduction therapy (SRT) has transformed the treatment landscape by targeting the underlying metabolic defect and mitigating systemic inflammation. Early diagnosis and timely initiation of therapy are essential to prevent irreversible organ damage and improve long-term outcomes. This review provides an integrated hematological perspective on GD, highlighting its pathophysiological basis, clinical manifestations, and diagnostic challenges through a representative real-world clinical case. By linking biological mechanisms to practical diagnostic reasoning, the review aims to facilitate earlier recognition of GD in hematology practice and ultimately improve patient outcomes.

## Introduction

Gaucher disease (GD), first described by Philippe Charles Gaucher in 1882, is the most prevalent autosomal recessive lysosomal storage disorder (LSD) within the sphingolipidoses (Table 1) and holds particular relevance in hematology [1]. Anemia, thrombocytopenia, and splenomegaly are frequent initial signs that lead to hematology referral, placing hematologists at the frontline of diagnosis [2]. Yet, GD is far from uniform, ranging from the non-neuronopathic type 1 (GD1) to the acute neuronopathic types 2 (GD2) and chronic neuronopathic type 3 (GD3). Globally, GD affects approximately 1/40.000–60.000 individuals, with a slightly higher prevalence in Europe and North America, without significant sex-related differences [3, 4]. However, the incidence is markedly higher among individuals of Ashkenazi Jewish ancestry, where it may reach as high as 1 in 800 live births [5].

**Table 1:** Genetic and clinical overview of sphingolipidoses

Disease	Enzyme Deficiency (gene)	Stored material	Inheritance	Clinical types/forms
Gaucher disease	β-glucocerebrosidase (*GBA1*)	Glucocerebroside	AR	Type 1, 2 and 3
Niemann-Pick	Sphingomyelinase (*SMPD1*)	Sphingomyelin	AR	Type A/B
	Lipid transport (*NPC1*/*NPC2*)	Cholesterol, sphingolipids	AR	Type C
GM_1_ gangliosidosis	β-galactosidase (*GLB1*)	GM_1_ ganglioside	AR	Type 1 (infantile); type 2 (late infantile/juvenile); type 3 (adult/chronic)
GM_2_ gangliosidosis	β-hexosaminidase A (*HEXA*)	GM_2_ ganglioside	AR	Tay-Sachs disease
	β-hexosaminidase A and B (*HEXB*)	GM_2_ ganglioside	AR	Sandhoff disease
	GM2 activator protein (*GM2A*)	GM_2_ ganglioside	AR	GM_2_ activator deficiency
Krabbe disease	Galactocerebrosidase (*GALC*)	Galactocerebroside	AR	Infantile / Juvenile / Adult
Fabry disease	α-galactosidase A (*GLA*)	Gb3	XR	Classic /late-onset
MLD	Arylsulphatase A (*ARSA*)	Sulphatides	AR	Late-infantile / Juvenile / Adult
*AR* autosomal recessive, *BM* bone marrow, *Gb*3 globotriaosylceramide, *H/S* hepato-splenomegaly, *MLD* metachromatic leukodystrophy, *NS* neurologic sign, *XR* X-linked recessive				

To date, substantial unmet needs persist, including impaired health-related quality of life, economic burden, frequent misdiagnosis, and diagnostic delays [6, 7]. An integrated understanding of GD requires linking its molecular pathogenesis with the hematological manifestations that often dominate clinical presentation. This review provides a hematologist-oriented perspective on GD, examining how GBA1-related metabolic and immune dysregulation translates into cytopenias, iron metabolism abnormalities, and clonal lymphoid disorders. Emphasis is then placed on the hematologic diagnostic workup and on practical strategies for early recognition and management, illustrated through a real-world representative clinical case.

### Clinical case

A 35-year-old man with β-thalassemia trait was initially referred to the Hematology Unit for evaluation of isolated mild chronic thrombocytopenia (100 × 10⁹/L). At that time, investigations for secondary causes were negative and the thrombocytopenia was attributed to immune thrombocytopenia. The patient was subsequently lost to hematologic follow-up. Ten years later, he returned to our attention after routine laboratory testing revealed a marked worsening of thrombocytopenia. At re-evaluation, laboratory assessment showed preserved hemoglobin levels (14.1 g/dL) but severe thrombocytopenia (32 × 10⁹/L). Liver function tests and serum protein electrophoresis were within normal limits, while serum ferritin was markedly elevated (1127 ng/mL). Abdominal ultrasonography demonstrated splenomegaly (longitudinal diameter 181.5 mm) and hepatomegaly.

At first glance, a clonal hematologic malignancy was considered in the differential diagnosis. Bone marrow aspirate showed an increased population of large histiocytic cells with fibrillary cytoplasm and eccentric nuclei, morphologically consistent with Gaucher cells. Bone marrow biopsy revealed hypercellularity (90%) with diffuse infiltration by CD68-positive histiocytes and no evidence of reticulin fibrosis.

In this clinical context, the combination of thrombocytopenia, splenomegaly, hyperferritinemia, and the presence of Gaucher-like cells on bone marrow examination raised suspicion for GD. Enzymatic and biomarker assessment was therefore performed on dried blood spot (DBS): β-glucocerebrosidase activity was markedly reduced (0.7 nmol/h/mL; pathological range 0.2–2.5 nmol/h/mL). Molecular analysis of the *GBA1* gene identified compound heterozygosity for the pathogenic variants c.508 C > T, p.R170C (R131C) and c.1226 A > G, p.N409S (N370S), confirming the diagnosis of non-neuronopathic GD. Skeletal involvement was evaluated by whole-body magnetic resonance imaging (MRI). Bone involvement was quantified using the bone marrow burden (BMB) score derived from lumbar spine and femoral MRI sequences, yielding a low score (4/16) consistent with mild skeletal disease.

Substrate reduction therapy (SRT) with eliglustat was initiated as first-line disease-modifying therapy. CYP2D6 genotyping confirmed extensive metabolizer status, allowing standard dosing of 84 mg twice daily. Early biochemical response was observed, with glucosylsphingosine (Lyso-Gb1) levels decreasing by approximately 50% within the first year of therapy paralleled by a progressive hematological response, with platelet counts increasing to approximately 100–110 × 10⁹/L. Ferritin levels decreased to 380 ng/mL, consistent with reduced macrophage activation. Follow-up skeletal assessment with MRI demonstrated stable marrow infiltration, with BMB score decreased at 2/16. At long-term follow-up, no new skeletal lesions or disease progression were observed. Treatment was well tolerated, with no clinically significant adverse events or need for dose modification. The sustained improvement in hematological parameters and disease biomarkers supported continued eliglustat therapy.

## Hematological consequences of *GBA1* mutations

### Mutational background

GD is caused by biallelic pathogenic variants in the *GBA1* gene, located on chromosome 1 (1q21) and encoding for glucocerebrosidase (GCase), a lysosomal enzyme also known as glucosylceramidase or acid β-glucosidase (EC 3.2.1.45). GCase catalyzes the cleavage of glucosylceramide (GlcCer) into ceramide and glucose, a critical step in maintaining lipid homeostasis within the lysosome [8]. Normally, a small fraction of GlcCer is also deacylated by lysosomal acid ceramidase into Lyso-Gb1 (or Lyso-GL1), a highly bioactive and cytotoxic lysolipid [8].

To date, over 500 *GBA1* mutations have been identified, including missense and nonsense variants, splice-site defects, and complex genomic rearrangements [9]. Because early Gaucher literature used a protein numbering system that excluded the signal peptide, several variants have dual nomenclature (e.g., N370S corresponding to N409S and R131C corresponding to R170C in HGVS notation) [10]. In this review, the historical nomenclature commonly used in Gaucher literature is retained for consistency.

These variants primarily impair the enzyme’s function, through alterations in its structure, stability, or expression, resulting in the accumulation of GlcCer [8]. Common pathogenic variants include N370S, L444P, 84GG, IVS2 + 1G > A, and RecNcil, the latter arising from recombination events between *GBA1* and its highly homologous pseudogene, *GBAP1*, located downstream [11–13]. A genotype–phenotype correlation has been recognized: the presence of N370S, either in homozygosity or compound heterozygosity, is predominantly associated with GD1, whereas the L444P mutation is more frequently linked to neuronopathic forms, particularly GD3, albeit with considerable clinical heterogeneity [14].

### Protein misfolding and endoplasmic reticulum stress

Newly synthesized glucocerebrosidase (GCase) normally undergoes folding in the endoplasmic reticulum (ER) and is subsequently trafficked to the lysosome, where it hydrolyzes glucosylceramide (GlcCer) [15]. In GD, many mutant GCase proteins display defective folding and are retained in the ER, where they are targeted for proteasomal degradation through ER-associated degradation pathways, reducing the amount of enzyme reaching the lysosome [16]. Accumulation of misfolded GCase induces chronic ER stress and activation of the unfolded protein response (UPR), a compensatory pathway that attempts to restore proteostasis but may also disrupt cellular homeostasis by promoting calcium release and oxidative stress [17]. These alterations can impair mitochondrial function and further amplify cellular stress responses, contributing to disease pathology [18, 19]. In neuronal cells, reduced GCase activity and accumulation of glucosylceramide also promote α-synuclein aggregation, while α-synuclein aggregates in turn impair GCase trafficking and activity, creating a pathogenic feedback loop that links GD to Parkinsonian neurodegeneration [20].

### Inflammation resulting from metabolic dysfunction

**Fig. 1:**
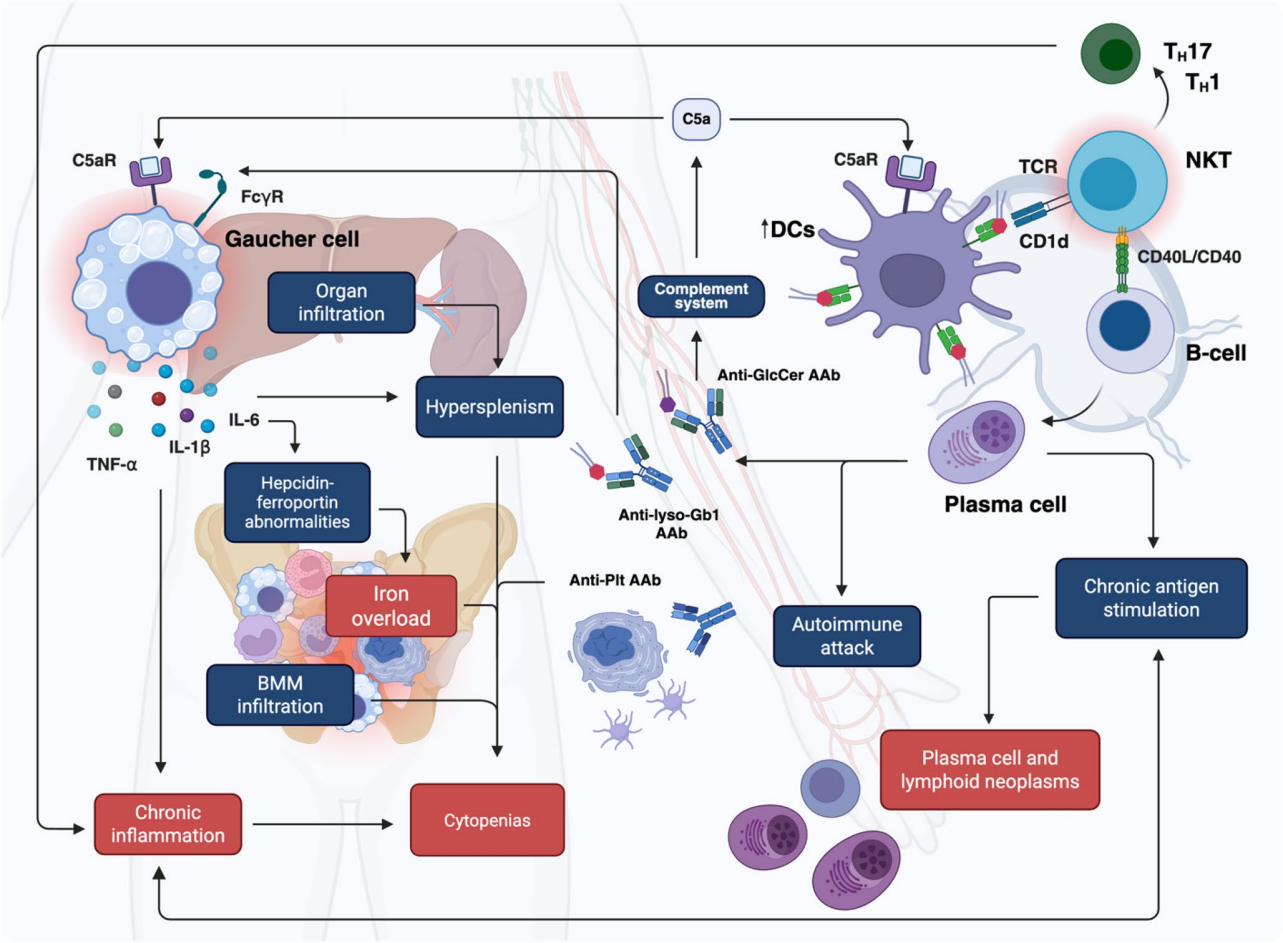
Immunopathogenesis of hematologic involvement in Gaucher disease (GD). Gaucher cell accumulation drives organomegaly and cytopenias through mechanical displacement and a sustained pro-inflammatory milieu. Secreted cytokines (IL-6, TNF-α, IL-1β) amplify immune activation. Impaired clearance of GlcCer and Lyso-Gb1 enhances APC recruitment and stimulates NKT, B, and T_H_1/T_H_17 cell responses. Anti-GlcCer AAb engage FcγR and activate the C5a–C5aR axis, further promoting inflammation. Immune dysregulation may also foster anti-Plt AAb production. Chronic antigenic stimulation likely underlies plasma cell and lymphoid neoplasms. Created in BioRender. Costa, A. (2026) https://BioRender.com/xilqzza. Abbreviations: AAb, autoantibody; APC, antigen-presenting cell; C5a-R, C5a receptor; DC, dendritic cell; FcγR, Fc gamma receptor; GCase, β-glucocerebrosidase; GlcCer, glucosylceramide; IL-1β, interleukin-1 beta; Lyso-Gb1, glucosylsphingosine; MGUS, monoclonal gammopathy of undetermined significance; MM, multiple myeloma; NKT cell, natural killer T cell; T_H_1, T helper type 1 cell; T_H_17, T helper type 17 cell; TNF-α, tumor necrosis factor alpha

Similar to other monogenic autoinflammatory syndromes such as VEXAS syndrome [21], GD underscores the interplay between metabolism, hematopoiesis, and immune regulation. Macrophage transformation into Gaucher cells and their infiltration of reticuloendothelial organs represent a central feature of disease biology [22]. For instance, splenic enlargement largely reflects the progressive accumulation of lipid-laden macrophages, although inflammatory and hyperplastic responses of the reticuloendothelial system also contribute to the overall increase in organ volume (Fig. 1) [23].

Although most of the molecular pathways described in this section have been characterized primarily in experimental or animal models, immune alterations in GD appear to extend beyond the simple accumulation of lipid-laden macrophages and involve a broader dysregulation of the monocyte–macrophage compartment. In this context, monocytes may acquire pro-inflammatory and antigen-presenting properties and contribute to the generation of Gaucher macrophages, promoting vascular obstruction and tissue injury through inflammatory and procoagulant mechanisms [24–26].

Gaucher cells display features consistent with alternatively activated (M2-like) macrophages, although current evidence suggests a complex polarization state involving both M1 and M2 pathways [26–28]. This dysregulated macrophage compartment is associated with increased production of pro-inflammatory cytokines, including IL-1β, IL-6, IL-8, TNF-α, and macrophage colony-stimulating factor, which contribute to hematologic and skeletal complications and may also influence the altered iron metabolism observed in GD [29]. Among macrophage-derived biomarkers, chitotriosidase (ChT) and CCL18 are widely used indicators of disease activity [30].

Macrophages from GD patients also exhibit defective efferocytosis due to impaired phagosome–lysosome fusion, leading to the persistence of apoptotic debris and prolonged exposure of autoantigens, which may contribute to the frequent detection of autoantibodies [31, 32]. Crosstalk between innate and adaptive immunity further amplifies this inflammatory environment. Activation of the C5a–C5aR1 axis [33, 34] and dendritic cell activation promote T-cell stimulation and cytokine production, reinforcing systemic immune dysregulation [33, 34].

### Gaucheromas as a tissue expression of macrophage dysfunction

Macrophage dysfunction in GD may lead to the formation of tumor-like aggregates of Gaucher cells, termed gaucheromas. These lesions may occur in a variety of anatomical sites including liver, spleen, bone, and even orbital or intracranial locations [35, 36]. While histologically benign and lacking metastatic potential, these lesions can exert significant local effects, including anatomical compression, skeletal deformities, and chronic pain [37]. Their pathogenesis remains incompletely understood, but Grabowski et al. [25] proposed a dynamic model wherein ongoing monocyte recruitment and macrophage polarization drive lesion development and progression [25, 38]. Alternatively activated M2 macrophages appear central to this process. Subclassified into M2a, M2b, M2c, and M2d, they perform diverse roles in tissue repair, immunoregulation, and angiogenesis [39, 40]. Early phase development is marked by a predominance of M2a and M2c macrophages expressing the mannose receptor (MMR), localized near vascular structures [25]. With disease progression, lysosomal dysfunction and impaired efferocytosis promote a shift toward MMR-negative M2b macrophages, which contribute to the formation of a poorly vascularized fibrotic core. Simultaneously, M2d macrophages at the lesion margins stimulate angiogenesis, favoring centripetal expansion [25, 37, 41]. This evolving macrophage milieu may explain the therapeutic resistance of established gaucheromas [42].

### Cytopenias, hyperferritinemia and coagulation abnormalities

Hematological abnormalities are among the most common manifestations of GD and often represent the initial clue to diagnosis. Cytopenias, in particular, are frequent and multifactorial [43]. In most cases, they are secondary to hypersplenism, but in others, significant infiltration of the bone marrow by Gaucher cells directly impairs hematopoiesis [29]. Mechanistic insights from the hematopoietic-specific VavCre 129 GD murine model have shown a reduction in myeloid and erythroid progenitor populations, despite preserved peripheral blood counts, thus mirroring the often delayed onset of cytopenias in patients [29]. These findings suggest the presence of early hematopoietic stress, initially masked by compensatory responses such as marrow hypercellularity or extramedullary hematopoiesis, which eventually become insufficient in the context of progressive substrate accumulation and chronic inflammation [29]. Occasionally, marked or rapidly progressive thrombocytopenia may result from an autoimmune mechanism, which has been described in association with GD. Bleeding manifestations may also occur despite platelet counts > 100 × 10⁹/L and normal coagulation profiles, suggesting qualitative platelet dysfunction. In such cases, impaired platelet adhesion due to excess GlcCer has been proposed, although the precise molecular mechanisms remain incompletely defined [44].

Iron overload is a well-documented feature of metabolic disorders, including GD. However, hyperferritinemia in GD does not reflect true parenchymal hepatic iron overload, but rather represents a marker of macrophage activation and chronic inflammatory signaling within the reticuloendothelial system [45]. The chronic low-grade inflammation of GD sustained by the upregulation of cytokines such as IL-6 and IL-10 may induce increased expression of hepcidin, levels of which are elevated in GD patients [46]. Hepcidin, in turn, inhibits ferroportin activity, thereby limiting cellular iron efflux and promoting intracellular iron retention. Additionally, increased splenic turnover of red blood cells contributes to an elevated iron load and hepcidin dysregulation [47]. It has been proposed that, in the early stages of disease, pro-inflammatory M1 macrophages may act as functional reservoirs for sequestered iron, as seen in other chronic inflammatory states [45]. However, under persistent inflammatory stimulation, a compensatory anti-inflammatory response emerges, with polarization towards the M2 phenotype. This transition facilitates iron release into surrounding tissues, contributing to oxidative stress and potential organ damage [45].

Coagulation abnormalities have been reported in several studies, including reduced levels of specific clotting factors [43]. These changes may reflect subclinical hepatic dysfunction, although alternative mechanisms may also contribute, including GlcCer accumulation itself or circulating antiphospholipid antibodies. Notably, in a cohort of 27 GD patients, reduced factor levels were documented even in the absence of biochemical liver abnormalities, supporting a multifactorial origin [48]. The contribution of hypersplenism to the bleeding diathesis is further supported by reports of improved coagulation profiles after splenectomy, including increased markers of coagulation and fibrinolysis [49]. However, findings remain inconsistent across studies; in some cases, factor levels remained depressed post-splenectomy, with variability among individual factors [48].

### Chronic inflammatory stimulation and clonal lymphoid disorders

Cumulative evidence indicates an increased risk of neoplasms in patients with GD, including a threefold incidence of hepatic and renal tumors, likely related to chronic inflammation, lipid accumulation, and long-standing immune dysregulation associated with lysosomal dysfunction [50, 51]. Among hematological malignancies, GD patients are at significantly elevated risk for monoclonal gammopathy of undetermined significance (MGUS), multiple myeloma (MM), and B-cell neoplasms. Specifically, the risk of non-Hodgkin lymphoma (NHL) has been found to be approximately three times higher than in the general population. A report by Rosenbloom et al. [52] on 2123 GD1 patients from the International Collaborative Gaucher Group (ICGG) Registry documented 69 cases of hematological malignancies, including 28 NHL and 22 MM cases. A prospective Italian case series identified MGUS in one out of 150 patients with GD1 [53], while a multicenter study estimated a prevalence of 7% among patients with GD under the age of 60, which is considerably higher than that observed in the general population [52]. Genetic factors influence the risk of gammopathy; patients homozygous for the p.Asn409Ser variant appear to exhibit a lower incidence of both MGUS and polyclonal gammopathy compared to those with other *GBA1* genotypes [52]. However, an analysis of a national French registry showed that the only independent predictor of MGUS in GD was older age at diagnosis, while sex, splenectomy, disease-specific treatment, genotype, and initial clinical features were not significantly associated [54].

Although the risk of MGUS is increased, no significant differences have been observed in the rate of progression to MM, with a ten-year progression rate of 8% compared to 10% in the general population [52, 55]. Nevertheless, the relative risk of developing MM is markedly elevated, ranging from 5.9 to 51.1 compared to the general population [56–58]. Recently, the CHAGAL multicenter cohort of 1004 patients with either smoldering or overt MM identified 14 cases (0.9 per 1.000) with GD, a rate approximately 100 times higher than the estimated prevalence in the Italian general population (0.009 per 1,000) [59]. Among these, 12 patients carried a single heterozygous mutation in the *GBA1* gene (commonly N370S or L444P), while the remainder exhibited compound or double heterozygosity.

The pathogenesis of lymphoid disorders in GD is not yet fully elucidated, although various immunopathogenic mechanisms have been proposed (Fig. 1). Central to these is the sustained and elevated production of cytokines that promote B-cell activation and survival, such as IL-1β, TNF-α, IL-6, and IL-10 [60]. In GD-associated MGUS/MM, the monoclonal immunoglobulin frequently recognizes Lyso-Gb1. Notably, reactivity to Lyso-Gb1 has also been observed in approximately one-third of sporadic MGUS/MM cases, suggesting that persistent antigenic stimulation mediated by Lyso-Gb1 may underlie B-cell clonal expansion [61].

Substrate reduction strategies targeting the accumulation of lysolipids have demonstrated therapeutic potential. In murine models, lysolipid depletion mitigated gammopathy [61]. Furthermore, dysfunction within the natural killer T-cell compartment, particularly an imbalance between type I and type II NKT cells, has emerged as a contributing factor. Type II antigen-specific NKT cells, reactive to β-glucosylsphingosine or β-glucosylceramide 22:0, exhibit a T follicular helper-like phenotype, thereby promoting B-cell activation. In vivo administration of βGL1-22 or Lyso-Gb1 induces expansion of these type II NKT subpopulations, resulting in germinal center hyperactivity, hypergammaglobulinemia, and the production of lipid-reactive autoantibodies. These findings establish a mechanistic link between glucosylceramide accumulation, NKT-cell activation, and antigen-driven B-cell dysregulation [62, 63].

## The hematologist’s role in the diagnostic workup

### Non-neuronopathic GD

GD1 accounts for the vast majority (90–95%) of cases and holds particular relevance for hematologists [3]. Despite its classical designation as a non-neuronopathic, adult-onset disorder, GD1 exhibits a bimodal age distribution, with incidence peaks around 10 and 30 years of age [14].

Thrombocytopenia, splenomegaly, and elevated ferritin levels often represent the earliest manifestations and should prompt consideration of GD in the differential diagnosis [64]. Splenomegaly is present in over 90% of patients at diagnosis, with approximately 30% exhibiting spleen volumes ≥ 15 times normal, particularly in those with N370S/84GG genotypes [14]; such massive enlargement is rare in N370S homozygotes, while intermediate volumes (5–15× normal) are seen in 55% of patients with mixed or undefined genotypes [14]. Hepatomegaly occurs in 60–80% of cases, generally mild to moderate; values ≥ 2.5× normal are uncommon and typically observed in children or those with severe genotypes [14]. Acute complications, including splenic infarction or spontaneous rupture, are rare but constitute hematological emergencies [65].

**Fig. 2:**
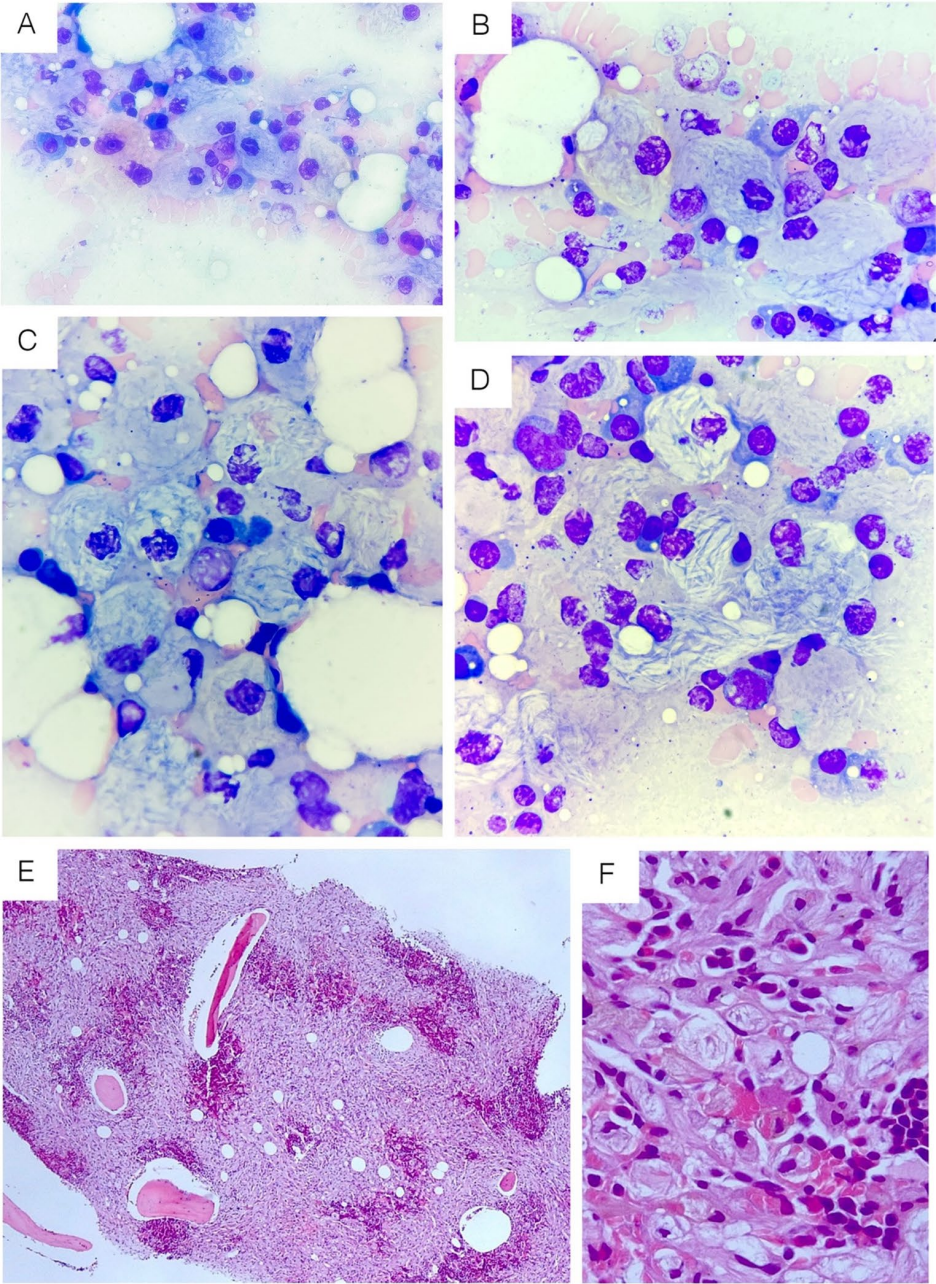
Cytological and histopathological features of Gaucher cells in bone marrow**.** Bone marrow smear showing large macrophages with eccentric nuclei and abundant fibrillary cytoplasm giving the typical “crumpled tissue paper” appearance (A, B, C, D); trephine biopsy reveals cluster of Gaucher cells infiltrating the marrow space and replacing normal hematopoiesis (E, F)

Hematological involvement includes anemia and thrombocytopenia, most often secondary to hypersplenism or marrow infiltration by Gaucher cells (Fig. 2). Leukopenia is rarely reported, and with the exception of splenectomized individuals, patients with GD have not been shown to have an increased risk of infection-related mortality [66]. According to ICGG data, anemia is observed in only 20–30% of cases at diagnosis, while moderate thrombocytopenia (60–120 × 10⁹/L) is present in 50–70% of patients [14]. A bleeding diathesis, typically mucocutaneous and mild, is reported in up to 20% of cases. Coagulation abnormalities, including prolonged PT/aPTT and deficiencies in factors V, X, and II, have also been described [43]. Hyperferritinemia with normal transferrin saturation is another frequent finding in GD1 and has been shown to correlate with disease severity and response to therapy [67].

Skeletal involvement is frequent at diagnosis and constitutes a major source of morbidity and reduced quality of life [68]. Skeletal manifestations may remain clinically silent for prolonged periods and are sometimes detected incidentally during imaging studies [69]. The clinical spectrum includes chronic diffuse or localized bone pain, acute infarctions of the metaphysis or epiphyses, avascular necrosis, joint collapse, pathological fractures, and vertebral compression secondary to osteopenia or osteoporosis [70]. Bone crises are reported in 15–22% of non-splenectomized patients and in up to 55% of splenectomized patients [71, 72]. When symptomatic, osteonecrotic lesions can precipitate acute bone crises, occasionally accompanied by fever and leukocytosis, which may mimic osteomyelitis, particularly in children [71].

Pulmonary involvement, though less common, is clinically relevant and may result from alveolar or interstitial infiltration by Gaucher cells, particularly in splenectomized patients. Contributing factors include vertebral deformities, pulmonary fibrosis, and pulmonary hypertension, most frequently observed in splenectomized individuals or in the context of hepatopulmonary syndrome secondary to cirrhosis [72]. Renal involvement is exceptional, with recent data showing no significant impairment in renal function [73].

Gaucheromas represent a rare but clinically relevant manifestation of GD. They are most commonly observed in types 1 and 3 and typically involve the liver, spleen, lymph nodes, and bones [37]. When located in the paravertebral region, extraosseous extension can lead to radiculopathies or, in more severe cases, acute spinal cord compression necessitating prompt neurosurgical intervention [39]. In a cohort of 74 patients with GD1 receiving ERT, imaging studies identified presumed gaucheromas in seven individuals, with lesions affecting the spleen, liver, and bone [37]. Further data from a Dutch cohort reported the presence of gaucheromas in nine out of forty patients with GD1 [38].

Although GD1 is traditionally defined as non-neuronopathic, emerging evidence indicates secondary neurological manifestations, including peripheral neuropathy and an increased risk of parkinsonism [74]. Variants in the *GBA1* gene have been associated with a higher risk of Parkinson’s disease and Lewy body dementia, even among heterozygous carriers, implicating the underlying enzymatic defect in the pathogenesis of neurodegeneration. Therefore, GD should be suspected in patients presenting with cytopenias and Parkinson’s disease, particularly when characterized by early onset, rapid symptom progression, cognitive decline, and a higher prevalence of non-motor symptoms compared to individuals with sporadic forms of the disease [75]. Nonetheless, most patients with GD1 remain neurologically asymptomatic, suggesting the involvement of additional, yet unidentified, genetic or environmental cofactors [25]. Unlike the neuronopathic forms GD2 and GD3, GD1 is typically limited to astrogliosis without evident neuronal loss or widespread glial infiltration [76].

### Neuronopathic GD

The neuronopathic forms of GD, classified as GD2 and GD3, are distinguished by primary and progressive neurological involvement. Although they share some systemic features with GD1, and some authors have proposed that neurological involvement may occur across all disease types, their natural history, clinical severity, and prognosis are markedly different.

GD2 is a rare form with an early onset and rapidly fatal course, characterized by severe neurological involvement from the first months of life [77]. The hematological phenotype is similar to that of GD1 in terms of splenomegaly, cytopenias, and bone marrow infiltration but is often more pronounced and progressive, with a frequent need for transfusion support as early as the neonatal period [77]. The presence of hematological abnormalities, splenomegaly, and ocular movement disorders should prompt the hematologist to consider neuronopathic GD [25]. However, the rapid progression of neurological symptoms typically limits therapeutic intervention, with a generally fatal outcome before the age of two [77].

The chronic neuronopathic GD3 exhibits an extremely heterogeneous clinical expression. From a hematological perspective, patients with GD3 typically present with significant visceral and hematological disease [78]. According to the ICGG registry, anemia was present in 62.6% of non-splenectomized patients at baseline, while 46.7% of splenectomized individuals were considered anemic [78]. At baseline, liver volumes were elevated in both non-splenectomized and splenectomized patients; in non-splenectomized patients, the average spleen volume was approximately 35 times the normal value, with more than 90% of patients presenting with severe splenomegaly [78]. Gaucheromas may also occur in patients with GD3, although data on genotype or phenotype prevalence within this population are lacking. A distinctive pattern of gaucheroma presentation has been described, characterized by massive mesenteric lymphadenopathy with confluent nodal enlargement and potential progression to protein-losing enteropathy secondary to lymphatic obstruction [25]. Finally, the appearance of neurological signs over time, such as slow ocular saccades (typically along the vertical axis), epilepsy, ataxia, or cognitive decline, should raise suspicion of GD3 [25]. Some patients may remain neurologically asymptomatic for many years, necessitating prolonged clinical monitoring by the hematologist in close collaboration with the pediatric neurologist [14].

### Early identification and diagnostic algorithms

Early identification of GD in hematology practice remains challenging, largely due to its heterogeneous presentation and limited awareness among clinicians. Indeed, early clinical manifestations are frequently nonspecific, and patients are often initially evaluated for more prevalent hematologic conditions such as lymphoproliferative disorders or immune thrombocytopenia before GD is considered, leading to prolonged diagnostic pathways and multiple specialist consultations [79]. In a national survey involving 35 Italian Hematology Units, 18% of annual first visits concerned patients presenting with splenomegaly and/or thrombocytopenia, and in over 10% of these cases, a definitive diagnosis was not reached [80]. Moreover, a multinational study found that only 20% of hematologists considered GD1 in the differential diagnosis of patients with anemia, cytopenias, organomegaly, or bone pain [81]. Consistently, patient-reported data highlight the magnitude of this issue. In a survey conducted by the International Gaucher Alliance (IGA) among individuals affected by GD, 58% of respondents reported waiting more than one year from the onset of symptoms before receiving a definitive diagnosis [82].

Diagnostic algorithms have been proposed to facilitate early recognition. In an Italian cohort of 196 patients with splenomegaly and/or thrombocytopenia associated with at least one additional feature (anemia, bone pain, early-onset monoclonal or polyclonal gammopathy < 30 years, or previous splenectomy), application of the Mistry et al. algorithm [83] yielded a GD1 prevalence of 3.6% [80]. A subsequent study by the same group developed a predictive equation based on three routine laboratory parameters (platelet count, ferritin, and transferrin saturation) to estimate individual probability of GD1 [84].

More recently, data-driven approaches have further expanded this concept. Machine-learning models developed using large electronic health-record datasets have demonstrated high discriminative performance for identifying patients with GD and may detect the disease several years before the clinical diagnosis is formally established [79, 85]. In particular, features such as splenomegaly, thrombocytopenia, bone manifestations, and hyperferritinemia consistently emerge among the most informative predictors in these models, reinforcing the diagnostic relevance of the hematologic and visceral phenotype that typically prompts referral to hematologists [85]. These findings support the integration of structured clinical algorithms and data-driven tools to improve case detection and reduce diagnostic delay in adult patients presenting with otherwise unexplained cytopenias and splenomegaly.

### Diagnostic workup and differential diagnosis

Recent consensus recommendations emphasize that targeted screening for GD should be considered early in the diagnostic work-up of patients presenting with unexplained splenomegaly, thrombocytopenia (< 100 × 10⁹/L), or elevated ferritin levels, particularly when these findings coexist or are associated with anemia, bone manifestations, or monoclonal and polyclonal gammopathy [64, 86]. Table 2 summarizes the main disorders commonly included in the differential diagnostic work-up and often initially suspected in hematological settings.

**Table 2:** Comprehensive differential diagnosis of Gaucher disease in hematological settings: morphological, clinical and molecular discriminants

Feature	GD	ASMD	CML	HCL	PMF	SMZL
Genetics/driver	*GBA1* mutations	*SMPD1* mutations	*BCR::ABL1*	*BRAF* V600E	*JAK2*/*CALR*/*MPL* mutations	*KLF2*, *NOTCH2* mutations etc.
Epidemiology	1.5/100.000 live births; 1:800 in Ashkenazi Jews	~ 1:250.000 live births	~ 2 /100.000 per year	0.1–0.3/100.000 per year	0.22–0.99/100.000 per year	0.5–2.92/100.000 per year
Age at onset	Variable: childhood to adulthood in GD1; earlier in GD2/3	Infancy (type A); childhood (type B)	Any age, peak in middle-aged adults	Adults; rare before 30 years	Typically > 60 years	Older adults
Cytopenias	Anemia; thrombocytopenia; rare neutropenia	Anemia and thrombocytopenia	Leukocytosis and/or thrombocytosis; anemia may be present	Pancytopenia, often with monocytopenia	Anemia; variable leukocyte and platelet counts	Anemia, thrombocytopenia, sometimes lymphocytosis
PB smear	Possible pseudo-Gaucher cells or activated monocytes with wrinkled cytoplasms	Rarely, vacuolated lymphocytes	Marked leukocytosis with left shift; basophilia; myelocytes, metamyelocytes	Small to medium “hairy” lymphoid cells	Teardrop cells (dacrocytes); leukoerythroblastosis; nucleated RBCs	Small mature lymphocytes, sometimes villous
BM morphology	Gaucher cells cluster; normo-hypercellular marrow;	Foam cells, rare sea-blue histiocytes (not specific)	Marked hypercellular with myeloid hyperplasia; dwarf megakaryocytes	Hairy cells; hypocellular aspirate with frequent dry tap; reticulin often increased	Variable cellularity; dense reticulin and/or collagen fibrosis; atypical megakaryocytes (hypercromatic, bulbous or “cloud-like” nuclei); frequent dry-tap	Paratrabecular or nodular infiltration by small B-cells; possible plasmacytoid differentiation
Other features	Bone pain; neuro signs	Neuroregression, pulmonary disease	Fatigue, weight loss	Infections; vasculitic features	Splenic infarcts, extramedullary hematopoiesis	Possible autoimmune phenomena
Key tests	Reduced GCase activity; *GBA1* sequencing	Reduced ASM activity; *SMPD1* sequencing	*BCR::ABL1* by FISH/RT-PCR	Immunophenotyping (CD11c, CD25, CD103, CD123); *BRAF* mutation	Bone marrow morphology; mutation analysis	Flow cytometry (CD20+, IgM+, CD11c+/-), BM biopsy critical
Therapy	ERT and SRT	ERT (olipudase alfa)	TKIs	Purine analogues; BRAF inhibitors	JAK inhibitors, allo-HSCT in selected cases; supportive therapy	Rituximab ± chemotherapy; splenectomy in selected
ASMD, acid sphingomyelinase deficiency; allo-HSCT, allogeneic hematopoietic stem cell transplantation; BM, bone marrow; CML, chronic myeloid leukemia; ERT, enzyme replacement therapy; FISH, fluorescence in situ hybridization; GCase, glucocerebrosidase; GD, Gaucher disease; HCL, hairy cell leukemia; MPN, myeloproliferative neoplasm; PB, peripheral blood; PMF, primary myelofibrosis; RBCs, red blood cells; RT-PCR, reverse transcriptase polymerase chain reaction; SMZL, splenic marginal zone lymphoma; SRT, substrate reduction therapy; TKIs, tyrosine kinase inhibitor						

**Fig. 3:**
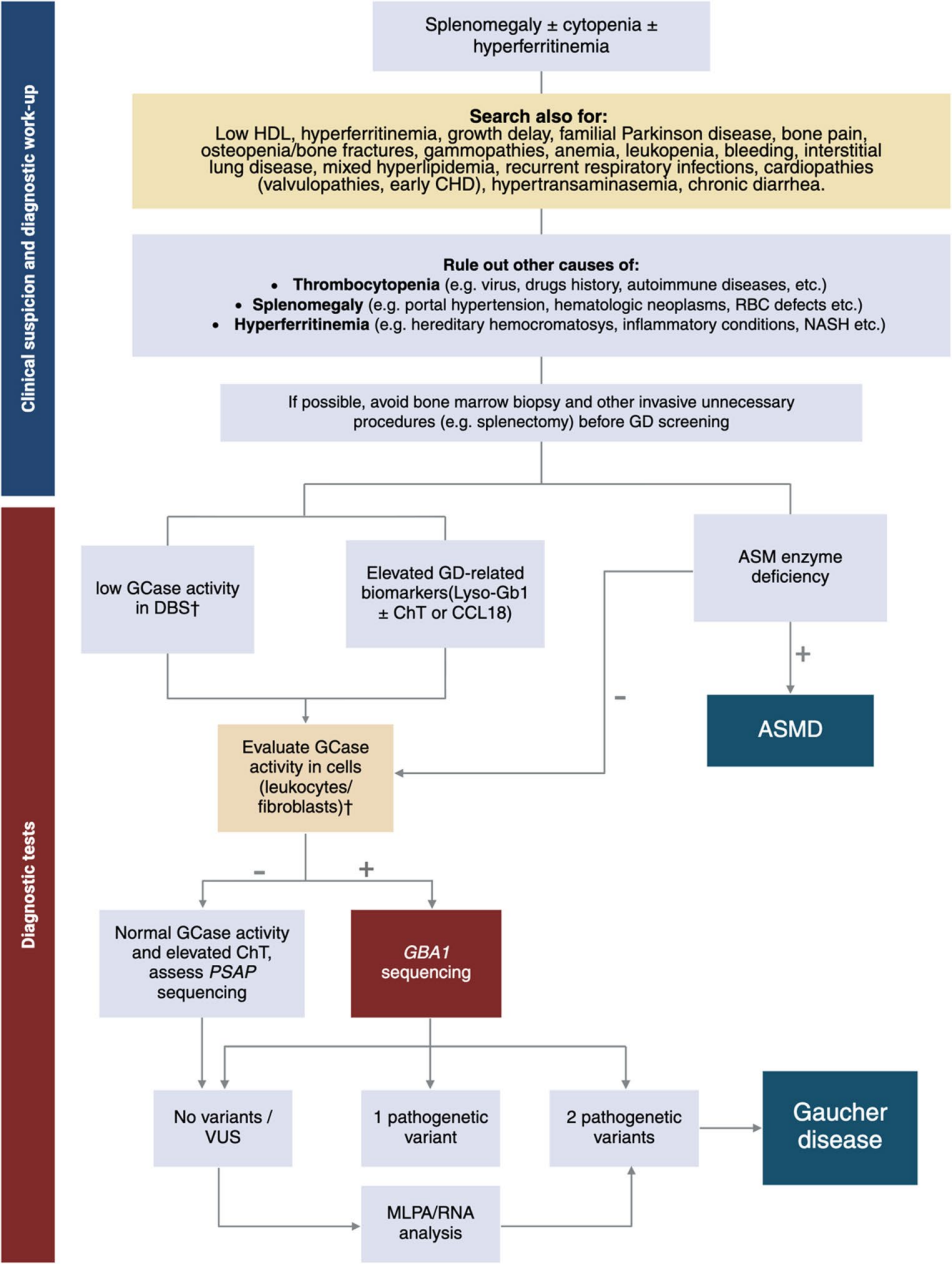
Diagnostic workup for Gaucher disease (GD), from clinical suspicion to molecular confirmation. Modified from [64, 83, 87]. Created in BioRender. Costa, A. (2026) https://BioRender.com/sp3shj9. Abbreviations: ASMD, acid sphingomyelinase deficiency; BM, bone marrow; ChT, chitotriosidase; DBS, dried blood spot; GCase, glucocerebrosidase; Lyso-Gb1, glucosylsphingosine; MGUS, monoclonal gammopathy of undetermined significance; MLPA, multiplex ligation-dependent probe amplification; NASH, non-alcoholic steatohepatitis; PSAP, prosaposin; RBCs, red blood cells; VUS, variant of uncertain significance.

Importantly, screening for GD should generally precede invasive diagnostic procedures (Fig. 3). Although suspicion of GD may arise incidentally during bone marrow examination performed for cytopenias or suspected marrow infiltration - where Gaucher cells can be identified morphologically [87] - bone marrow biopsy is not recommended as a primary diagnostic test, since enzymatic assays provide a more reliable and less invasive diagnostic approach [86]. Moreover, the absence of Gaucher cells in bone marrow does not exclude GD, and pseudo-Gaucher cells may occur in other hematologic conditions such as myeloma, leukemia, or myelodysplastic syndromes [83]. However, according to a recent Italian expert consensus, bone marrow evaluation may still be appropriate in selected clinical settings, particularly in cases of severe or rapidly progressive cytopenias, where alternative hematologic conditions must be urgently excluded [64].

Hyperferritinemia is a common laboratory abnormality in GD and frequently accompanies thrombocytopenia and splenomegaly [89]. In adult patients, ferritin levels exceeding 800 ng/mL should prompt further diagnostic evaluation, even when hyperferritinemia is the only abnormal finding [64]. In this setting, assessment of transferrin saturation is essential in the initial evaluation of hyperferritinemia to distinguish GD from hereditary hemochromatosis and other primary iron overload syndromes [64, 90].

Definitive diagnosis of GD is established by demonstrating deficient GCase enzymatic activity in peripheral blood cells, in combination with *GBA1* genotyping [89]. Enzymatic activity can be assessed in peripheral leukocytes, cultured fibroblasts, or using DBS. While DBS represents a minimally invasive and suitable method for screening, confirmatory testing in nucleated cells is recommended due to preanalytical variability [91]. Leukocyte-based assays must be performed within 24 h; cultured fibroblasts are preferred in transfused patients or in cases with borderline results. An enzymatic activity < 15% relative to controls is considered diagnostic [89]. A structured, multidisciplinary screening initiative conducted in Sardinia demonstrated that the integrated diagnostic pathway combining clinical “red flags”, DBS enzymatic assays, and second-tier biomarkers can facilitate earlier recognition of GD in real-world hematology practice [92].

Among biomarkers, Lyso-Gb1 is currently the most specific and informative biomarker for GD. Initially identified in the CNS of patients with neuronopathic GD, it has subsequently been detected in plasma, spleen, and liver [93, 94]. Measurement via DBS is accurate, with high sensitivity and specificity. Lyso-Gb1 levels correlate with disease burden and clinical parameters such as liver volume and bone density [95, 96] while correlation with *GBA1* genotype remains less clearly defined [97, 98]. Among others, ChT and CCL18 are widely used, though both present limitations. ChT, secreted by Gaucher cells, may be elevated up to 1000-fold, but inactivating *CHIT1* polymorphisms (e.g., dup24, p.Glu74Lys) may abolish or markedly reduce its activity [99, 100]. CCL18, produced by alternatively activated macrophages, is elevated in GD but lacks specificity, as it may also be increased in other inflammatory or storage disorders such as acid sphingomyelinase deficiency (ASMD), and atherosclerosis [89].

In this setting, prompt recognition and exclusion of ASMD is essential, underscored by the availability of ERT with olipudase alfa [101]. In contrast to GD, ASMD typically presents with progressive hepatic dysfunction, significant pulmonary involvement, and a profound pro-atherogenic dyslipidemia characterized by elevated LDL cholesterol, reduced HDL levels, and increased triglycerides. These features, particularly the pulmonary and atherogenic-related cardiovascular events, frequently converge as the principal determinants of morbidity and mortality [88].

A definitive diagnosis of GD requires identification of two pathogenic biallelic variants in *GBA1* [25]. Genetic testing is indicated in the presence of reduced GCase activity, particularly in patients with atypical phenotypes or discrepancies between enzymatic and biomarker profiles [25]. The standard method involves selective PCR amplification of the functional gene, followed by Sanger sequencing. However, this approach may fail to detect large deletions [102]. Next-generation sequencing (NGS) technologies, including targeted panels, whole exome sequencing (WES), and whole genome sequencing (WGS), offer valid alternatives but require specialized bioinformatic pipelines to avoid misalignment errors due to the near-identical sequence of the *GBAP1* pseudogene [25]. Some *GBA1*-specific NGS platforms can accurately identify both point mutations and recombinant alleles (excluding RecΔ55) [103, 104]. Conversely, inclusion of *GBA1* in standard, non-optimized panels may detect only point mutations and is unreliable for identifying recombinant or structural variants [105]. Emerging long-read single-molecule sequencing methods (e.g., PacBio SMRT) show promise for comprehensive locus resolution but remain limited to research settings. Additionally, MLPA testing is available for detecting deletions and recombinant alleles, though it lacks resolution to distinguish specific events such as L444P versus RecNciI [106].

## Hematological issues in the management and follow-up of GD

### Disease-modifying therapies in GD1

**Fig. 4:**
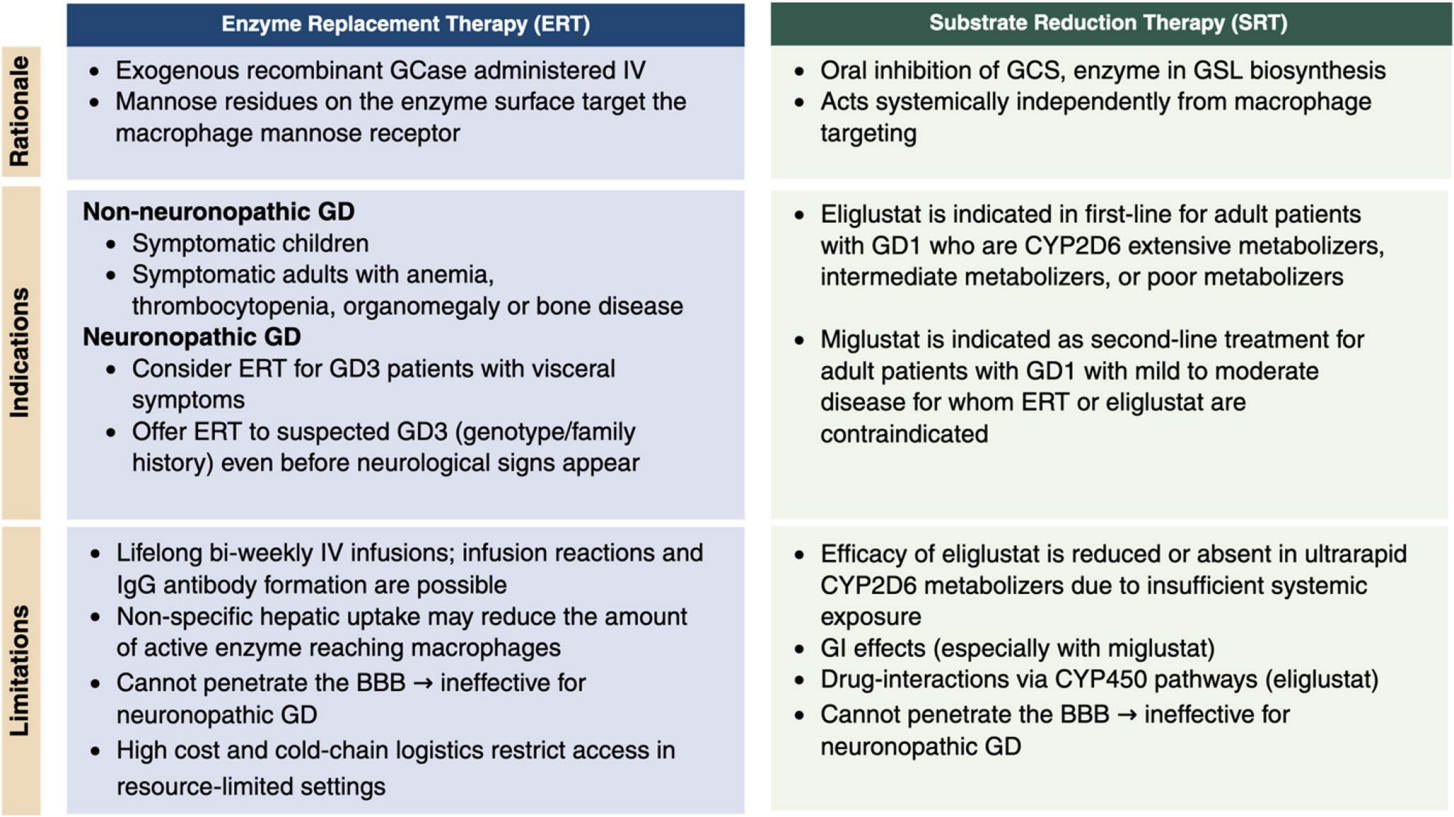
Comparative overview of enzyme replacement (ERT) and substrate reduction therapies (SRT) in Gaucher disease (GD): mechanisms, clinical indications, and limitations. Abbreviations: BBB, blood-brain barrier; GD1, Gaucher disease type 1; GD3, GD type 3; GCase, β-glucocerebrosidase; GCS, glucosylceramide synthase; GLS, glycosphingolipid; IV, intravenous

Over the past three decades, the therapeutic landscape of GD1 has shifted from a predominantly symptomatic approach to disease-modifying strategies that target the underlying metabolic defect (Fig. 4).

ERT represented the first effective intervention capable of altering the natural course of the disease by restoring functional glucocerebrosidase activity in affected target cells, particularly pathological macrophages. Since its introduction in the mid-1990s, ERT has demonstrated sustained efficacy, leading to progressive improvement in hematological parameters, reduction of organomegaly, and stabilization of skeletal disease in the majority of treated patients. Long-term observational studies indicate that more than 90% of patients achieve correction of anemia and significant improvement in thrombocytopenia after prolonged therapy, with durable responses maintained over decades of follow-up [107, 108].

SRT has subsequently expanded therapeutic options by targeting the synthesis of glycosphingolipids upstream of the metabolic defect. Eliglustat, approved in 2014, inhibits glucosylceramide synthase and has demonstrated efficacy in both treatment-naïve patients and those previously stabilized on ERT, resulting in sustained hematologic and visceral responses [109]. However, its use requires prior CYP2D6 pharmacogenetic testing to determine metabolizer status and careful assessment of potential drug–drug interactions, particularly with CYP2D6 inhibitors or substrates. In addition, eliglustat is contraindicated during pregnancy and lactation [110]. Tables 3 and 4 summarize key clinical trials of ERT [111–117] and SRT [96, 118–123] approved for GD1 management.

**Table 3:** Selected clinical trials on enzyme replacement therapy (ERT) for GD1

Trial	Study design	*N*. pts/cohort	Efficacy outcomes	Adverse events
Imiglucerase				
Grabowski et al. [111]	• Phase 3 • Randomized alglucerase vs. IMI	• 30 GD1 pts (7 pediatric)	• Comparable improvements in hematologic, organ volumes, biomarkers	• Higher ADA rate with alglucerase • No severe AEs
Kishnani et al. [112]	• Phase 4, open-label • Schedule q2w vs. q4w	• 95 GD1 pts • *n* = 33 in q2w; 62 in q4w cohort	• q4w dosing may be considered in stable adults on IMI ≥ 2 years	• Similar rates of TRAEs in both arms
Velaglucerase alfa				
Turkia et al. [113]	• Phase 3 • Randomized IMI vs. VELA	• 35 treatment-naïve GD1; 17 per arm	• Non-inferior hematologic and visceral outcomes • Comparable biomarker responses	• Predominantly mild/moderate TRAEs • ADA detected in VELA cohort
Pastores et al. [114]	• Open-label switch due to IMI shortage (NCT00954460)	• 211 GD1 pts • *n* = 6 treatment-naïve; 205 previously treated	• Hematologic and visceral stability maintained post-transition	• Infusion-related reactions in 13% • Rare development of non-NAb
Taliglucerase alfa				
Zimran et al. [115]	• Phase 3 (NCT00376168) • Randomized dose- comparison (30 vs. 60 U/Kg)	• 31 GD adult pts treatment-naïve	• Dose-dependent in improvements in hematologic, organ volumes, biomarkers	• Mild/moderate TRAEs; • No severe TRAEs
Pastores et al. [116]	• Phase 3, open-label switch (PB-06-002; NCT00712348)	• 31 GD1 • *n* = 26 adults; 5 children • Previously treated with IMI	• Stable hematologic and visceral disease parameters following switch from IMI	• Mild/moderate and transient TRAEs • No severe TRAEs • No discontinuations due to TRAEs
Pastores et al. [117]	• Phase 3 (PB-06-003; NCT00705939) • Adults GD from PB-06-001 or PB-06-002	• *n* = 26 GD treatment-naïve pts; 19 treatment-switched	• Improved hematologic indices and biomarker profiles • Maintenance of clinical stability in switched patients	• Mild/moderate transient TRAEs • No severe TRAEs
ADA, anti-drug antibody; AEs, adverse-events; BMD, bone-marrow density; ChT, chitotriosidase; GD1, Gaucher disease type 1; IMI, Imiglucerase; non-Nab, non-neutralizing antibodies VELA, velaglucerase alfa; TRAEs, treatment-related adverse events				

**Table 4:** Selected clinical trials on substrate reduction therapy (SRT) for GD1

Trial	Study design	*N*. pts/cohort	Efficacy outcomes	Adverse events
Miglustat				
Cox et al. [118]	• Open-label, 12 mos study	• 28 adult GD1 pts (7 with prior splenectomy)	• Reduction in organ volumes • Mild improvement in hematologic and biomarker	• Most commonly GI-AEs • Few discontinuations reported
Cox et al. [119]	• Phase 3b (NCT00319046) • Switch from long-term ERT to miglustat	• 42 adult GD1 pts previously stabilized on ERT	• Hepatic volume remained stable in most pts • Some pts experience progressive disease features	• Predictable tolerability profile • GI-AEs were most frequent cause of discontinuation
Eliglustat				
Lukina et al. [120]	• Phase 2 (NCT00358150) • Open-label, single-arm	• 26 GD1 pts	• Improvements in hematological indices, biomarkers, organ size and bone manifestations	• Mild/moderate transient TRAEs • No severe TRAEs • 3 asymptomatic NSVT
Mistry et al. [96, 121]	• Phase 3 ENGAGE trial (NCT00891202), • Randomized, double-blind, placebo-controlled	• 40 GD1 pts treatment-naïve • Eliglustat, *n* = 20; placebo, *n* = 20	• Improvements in hematological indices, biomarkers, organ size and bone manifestations compared to placebo arm	• 55% had mostly mild/moderate TRAEs • 2 severe TRAEs (AV block) • Common TRAEs: headache (10%), dizziness, diarrhoea, abdominal pain/distension
Charrow et al. [122]	• Phase 3 EDGE trial (NCT01074944) • Randomized, double-blind trial • QD vs. BID dosing (same total daily dose)	• 170 GD1 pts, CYP2D6-compatible	• Hematologic and visceral parameters maintained within targets in both group	• Similar AEs profile in both group • 4 severe TRAEs (arrhythmia; moderate-severe vasovagal syncope) • 4 discontinued (2%) for TRAEs • Slightly better tolerability in BID arm
Cox et al. [123]	• Phase 3 ENCORE trial (NCT00943111) • Open-label, non-inferiority trial • Switch from ERT (IMI) to eliglustat	• 160 GD1 pts on stable ERT • Eliglustat, *n* = 106; IMI, *n* = 54	• 85% in eliglustat vs. 94% in IMI cohort met composite endpoint† • Maintained Hb, Plt, organ volumes	• Common eliglustat TRAEs: diarrhea (5%), arthralgia (4%), weakness (4%), headache (4%)
*ERT* enzyme replacement therapy, *GI* gastrointestinal, *LTE* long-term extension, *NSVT* non-sustained ventricular tachycardia, *TID* three times daily; † Hb decrease not > 15 g/L, Plt count decrease not > 25%, spleen volume increase not > 25%, and liver volume increase not > 20%, in multiples of normal from baseline				

### Hematological monitoring during follow-up

From a hematological perspective, the follow-up of patients with GD requires a structured and multidisciplinary monitoring strategy aimed at evaluating disease progression, treatment response, and the emergence of new complications. Current recommendations suggest periodic clinical and laboratory reassessment at intervals of approximately 6–12 months, although more frequent evaluations may be required in patients with active disease or during therapeutic adjustments [124–127]. Routine hematological monitoring includes complete blood count with hemoglobin and platelet levels, together with clinical assessment for bleeding manifestations such as epistaxis, mucosal bleeding, or easy bruising [124–127]. When clinically indicated—particularly before invasive procedures—coagulation parameters should also be evaluated, as mild coagulopathy and platelet dysfunction may contribute to hemorrhagic complications in GD [128].

Biochemical surveillance also plays a central role. Disease-specific biomarkers derived from macrophage activation, particularly Lyso-Gb1, are currently considered the most informative markers for disease monitoring [94, 129]. Lyso-Gb1 levels correlate with disease burden and respond rapidly to both enzyme replacement therapy and substrate reduction therapy, making them particularly useful for assessing therapeutic efficacy [129]. Alternative biomarkers, such as chitotriosidase or CCL18, may also be measured; however, their interpretation may be limited by genetic polymorphisms or lower disease specificity [129].

Monitoring of visceral involvement is also essential. Serial assessment of liver and spleen size, preferably by MRI or, when unavailable, abdominal ultrasonography, is recommended every 1–2 years to evaluate treatment response and detect progressive organomegaly or focal complications [124–127]. In addition, periodic measurement of liver enzymes and iron parameters, including ferritin, may provide indirect information on disease activity and associated metabolic disturbances [124–127].

### Persistent cytopenias during treatment

Despite the remarkable hematological outcomes achieved with modern therapies, the persistence or new onset of cytopenias during treatment should not automatically be interpreted as therapeutic failure. Alternative causes must be actively investigated, including hypersplenism, autoimmune cytopenias, hepatic dysfunction, or the emergence of clonal hematologic disorders. Likewise, persistent thrombocytopenia may reflect immune-mediated platelet destruction or bone marrow pathology rather than inadequate metabolic control [108].

### Skeletal disease and long-term morbidity

Although hematological and visceral manifestations usually respond rapidly to treatment, skeletal disease tends to improve more slowly and may persist despite effective systemic therapy [130]. Bone infarctions, avascular necrosis, and chronic bone pain therefore remain major determinants of long-term morbidity in GD, underscoring the importance of early treatment initiation and regular imaging surveillance to detect irreversible skeletal complications [131]. Given the high prevalence of skeletal complications in GD, regular evaluation of bone involvement is a critical component of follow-up. Clinical assessment should address bone pain and functional limitation, while MRI allows detection of bone marrow infiltration, infarctions, or osteonecrosis [127]. In patients at risk of osteoporosis, dual-energy X-ray absorptiometry (DXA) is recommended at baseline and periodically thereafter [127].

### Monoclonal gammopathy and plasma cells disorders

The impact of GD-specific therapy on MGUS and progression to MM remains unclear. In a cohort of 79 patients treated with ERT, total immunoglobulin levels declined across isotypes, but monoclonal components remained unaffected [132]. Conversely, an observational study reported MGUS regression following ERT [133]. A more recent case described a patient with GD1 and smoldering MM who, after three years of imiglucerase, exhibited a reduction in monoclonal burden, including IgA and free λ light chains, with normalization of the κ/λ ratio and a decrease in plasma cell infiltration from 20 to 30% to 5–10% [134]. In the same report, a second patient receiving eliglustat showed resolution of cytopenias and a decline in disease markers.

### Gaucheromas and other difficult-to-treat complications

Gaucheromas present a therapeutic challenge, and no standardized approach currently exists. Escalation of ERT has been attempted with variable results. Eliglustat, due to improved tissue penetration, has shown reduction of lesions in selected cases, including when combined with ERT [135, 136]. Nonetheless, response is often limited by the fibrotic and poorly vascularized nature of these masses. Surgical resection may be indicated based on anatomical considerations [25]. Radiotherapy is rarely used, although it has been applied in isolated cases of GD-associated bone tumors [137].

### Unmet therapeutic needs in neuronopathic GD

Unlike GD1, neuronopathic GD remains largely refractory to current therapies, as neither ERT nor SRT crosses the BBB [138]. Although systemic manifestations may improve, neurological progression typically continues. Investigational approaches, including pharmacological chaperones (e.g., ambroxol), CNS-targeted delivery systems, gene therapy using adeno-associated virus (AAVs) or lentiviral vectors, and genome editing platforms such as CRISPR/Cas9, are under active development [139]. These novel strategies may ultimately offer viable therapeutic options for neuronopathic disease, where current treatments remain ineffective.

## Conclusions

GD serves as a paradigm of systemic pathology, in which a single genetic mutation can trigger a cascade of inflammatory responses and complex hematological dysfunctions. Recognizing the central role of the monocyte–macrophage system not only elucidates the disease’s pathophysiology but also compels a broader clinical perspective from the hematologist. A more informed and integrated diagnostic approach, grounded in clear biological knowledge, is crucial to improving patient outcomes and reducing the burden of delayed or missed diagnoses. 

## Data Availability

no new data were generated or analysed in this study. Data sharing is not applicable to this article.
